# *Beauveria bassiana* for the simultaneous control of *Aedes albopictus* and *Culex pipiens* mosquito adults shows high conidia persistence and productivity

**DOI:** 10.1186/s13568-019-0933-z

**Published:** 2019-12-21

**Authors:** Jin Yong Lee, Ra Mi Woo, Cheol Jun Choi, Tae Young Shin, Won Seok Gwak, Soo Dong Woo

**Affiliations:** 1 Department of Agricultural Biology, College of Agriculture, Life & Environment Science, Chungbuk National University, Cheongju, 28644 Republic of Korea; 2 Department of Agricultural Biotechnology, College of Agriculture & Life Science, Seoul National University, Seoul, 08826 Republic of Korea; 3 Department of Agricultural Biology, College of Agriculture & Life Sciences, Chonbuk National University, Jeonju, 54896 Republic of Korea

**Keywords:** *Beauveria bassiana*, *Aedes albopictus*, *Culex pipiens*, Simultaneous control, Mosquito adults

## Abstract

This study was conducted to determine the optimal entomopathogenic fungus for the simultaneous control of the adults of two mosquito species, *Aedes albopictus* and *Culex pipiens*. The pathogenicity and virulence against the two species of mosquitoes were evaluated by using 30 isolates of *Beauveria bassiana*, an entomopathogenic fungus isolated from Korea that has high thermotolerance and UV-B tolerance. Regarding pathogenicity, 23 isolates were pathogenic to *Ae. albopictus* and 12 isolates were pathogenic to *Cx. pipiens*; *Ae. albopictus* adults were more susceptible to *B. bassiana* than *Cx. pipiens* adults. Among the isolates, 6 isolates that were simultaneously pathogenic to the two species of mosquitoes were used to evaluate virulence and conidia productivity. *B. bassiana* CN6T1W2 and JN5R1W1 had higher virulence than the other isolates, and they were more virulent in *Ae. albopictus* than in*Cx. pipiens*. The conidia productivity of *B. bassiana* JN5R1W1 on millet grain medium was higher than that of *B. bassiana* CN6T1W2. Based on these results, *B. bassiana* JN5R1W1 was selected as the most efficient isolate for the simultaneous control of the two mosquito species. *B. bassiana* JN5R1W1 can be used effectively in the development of fungal insecticides to simultaneously control *Ae. albopictus* and *Cx. pipiens* adults with similar distribution areas.

## Introduction

Mosquitoes are the most important vectors of severe human viral diseases, such as malaria, dengue, chikungunya, Zika, West Nile, and urban yellow fever, in tropical and subtropical regions (Mayer et al. 2017; Weaver et al. 2018). Mosquitoes are considered to be the most important vector species in the world, not only because of their susceptibility to these diseases but also because they target more than one host during their lifecycle (Fernandes et al. 2018; Harrington et al. 2001). Among the mosquitoes, the Asian tiger mosquito, *Aedes albopictus* (Skuse) (Diptera: Culicidae), transmits many viral diseases, such as yellow fever, dengue and chikungunya. It is also believed to have the potential for Zika transmission among humans due to the host capacity of the Zika virus (Hochedez et al. 2006; Wong et al. 2013). In addition, the house (or northern house) mosquito, *Culex pipiens* (L.) (Diptera: Culicidae), is a temperate Eurasian nocturnal mosquito that has established populations throughout temperate North America (Farajollahi et al. 2011). *Culex pipiens* may be strong nuisance and mediate a number of infectious diseases, such as Japanese encephalitis, West Nile, and Rift Valley fever virus (Farajollahi et al. 2011). Moreover, since these mosquito species are present in areas that contain more than half of the world’s population, continuous mosquito control efforts are needed to prevent the prevalence of these diseases (Khaiboullina et al. 2018; Kraemer et al. 2015; Lounibos 2002). In addition, due to recent global climate anomalies, the distribution of these mosquito species and related arboviral diseases are spreading (Shuman 2011). Therefore, these mosquitoes are two major mosquito vectors whose importance is increasing globally. However, since there are no vaccines against these viruses, except for Japanese encephalitis, controlling the mosquitoes that spread them is a method to prevent a pandemic due to these diseases.

There are two methods for controlling mosquitoes: chemical and biological control. Chemical control has been widely used as a major strategy to control mosquito density. However, the side effects of synthetic chemical insecticides in the environment have become widely popular concerns (Hemingway and Ranson 2000; Liu 2015; Moyes et al. 2017). Over the past several decades, the use of synthetic chemical insecticides by agricultural and public health programmes have caused many problems, including insecticide resistance, environmental pollution, and toxicity to humans and non-target organisms (Ariee et al. 2001; Damalas and Eleftherohorinos 2011; Mužinić and Želježić 2018). Therefore, integrated control methods have been attempted to overcome these limitations, but new strategies have been demanded because the integrated methods have not been effective thus far (Patterson 2016). The most attractive method is biological control.

One of the most successful biological control methods is mosquito larval control using *Bacillus thuringiensis israelensis* (Bti) and *Lysinibacillus sphaericus* (Berry 2011). Larval control is effective; however, adult control is largely dependent on synthetic chemical insecticides and the control effect has not been achieved. Therefore, it is necessary to develop a new biological control method for adult mosquito control; the representative method is to use entomopathogenic fungi. Entomopathogenic fungi are already widely used in agricultural pest control and are considered harmless and safe to humans and other animals (Lacey et al. 2001, 2015). In particular, they have a great advantage of infecting insects, including mosquitoes, through contact with spores compared to other biological control agents that need to be ingested, such as bacteria, viruses and protozoa. Fungal insecticides using various entomopathogenic fungi have been widely developed for agricultural pests, but despite much effort in their development for mosquito control, authentic products have only recently been developed and require more research efforts (Buckner et al. 2017; Snetselaar et al. 2014).

Among the entomopathogenic fungi used in biopesticide development, *Beauveria bassiana* and *Metarhizium anisopliae* are the most widely used (Lacey et al. 2015). These fungi have a relatively wide host range and are regarded as environmentally friendly fungi; various biopesticides have been developed with these agents to control agricultural pests and vectors such as ticks, termites and house flies (Lacey et al. 2001, 2015). In particular, *M. anisopliae* has been studied in a variety of mosquito species, but *B. bassiana* has been rarely studied in mosquitoes (Huang et al. 2017; Scholte et al. 2004). Virulence and persistence together with the productivity of conidia is required to determine the potential of a fungus for further development as a successful biopesticide (Fernandes et al. 2008, 2015; Lee et al. 2015; Lopez-Perez et al. 2015; Muñiz-Paredes et al. 2017; Samish et al. 2014). In the past, the selection of fungi for the development of biopesticides was mainly focused on virulence, and they was difficult to put into practical use due to problems with the persistence and productivity of fungal conidia. Therefore, recently, a simultaneous evaluation method for virulence, spore persistence and productivity using a large number of fungi has emerged. To date, the selection of fungi against mosquitoes has also been conducted with a focus on virulence, making it difficult to develop successful products.

To develop a successful fungal insecticide for the simultaneous control of *Ae. albopictus* and *Cx. pipiens* mosquito adults, our study was conducted to select fungi with high virulence and high conidia persistence and productivity. For this purpose, the virulence against mosquito adults and the conidia productivity of *B. bassiana* isolates with high conidia persistence among 342 fungal isolates from Korea (Shin et al. 2013) was evaluated. The most effective fungal isolates were finally selected by evaluating the conidia productivity of the selected fungal isolates.

## Materials and methods

### Mosquitoes

*Aedes albopictus* and *Cx. pipiens* mosquitoes were obtained from a colony at the laboratory of Insect Ecology and Toxicology, Chungbuk National University, Korea, and have been cultured under laboratory conditions. A single generation of mosquito adults was reared by placing eggs in plastic trays containing water to which 1 g of fish food was added. Larval trays were covered with a netting material. Larvae were fed fish food daily. Pupae were manually removed and placed in 30 cm cubic net cages. Adult males were provided with 10% sucrose solution, while females were fed twice per week on the blood of white mice. All of the mosquitoes were maintained at 28 ± 2 °C and 75 ± 5% RH with a 12 h photoperiod.

### Fungi

One hundred nine isolates of *B. bassiana* from 342 entomopathogenic fungal strains isolated in Korea (Shin et al. 2013) were used. Fungal isolates were cultured on potato dextrose agar (PDA) plates for 14 days at 25 °C. Then, 14-day-old fungal conidia were collected by scraping the fungi from the PDA plates and resuspending the material in a 0.01% Tween-80 (Difco, USA) solution. The conidial suspension was vigorously stirred and filtered through cotton to remove the mycelial debris. The conidial concentration was determined using a haemocytometer.

### Thermotolerance and UV-B tolerance for conidial germination

To evaluate the thermotolerance for conidial germination, 100 μL of conidial suspensions (5 × 10^6^ conidia/mL) were transferred to sterile PCR tubes and immediately placed in a thermal cycler adjusted to 45 °C. Aliquots (20 μL) were removed after 4 h and plated (without spreading) on Sabouraud dextrose agar (SDA; Difco TM, USA) with 0.5 μg/mL of benomyl (95% active ingredient) (SDA-B) (Fernandes et al. 2008). Plates were incubated at 25 °C for 24 h before microscopic observation of germination. For the evaluation of conidial germination following UV-B irradiation, 20 μL of a conidial suspension (5 × 10^6^ conidia/mL) from each isolate was plated (without spreading) on SDA-B. Conidia were immediately exposed to irradiances of 0.2 J/cm^2^ in a UV irradiation chamber (Bio-Link-BLX-E254, Vilber Lourmat, France) at 25 °C (Fernandes et al. 2015). After exposure, the plates were incubated at 25 °C. Control conidial suspensions were not exposed to UV-B but were inoculated on SDA-B at 25 °C. The relative percent germination was calculated by comparing germination to untreated isolates. At least 100 conidia were examined for each treatment in every experiment.

### Bioassay

Fourteen-day-old fungal conidia were used for the bioassay. A conidial suspension with more than 90% viability was used for the bioassay. The viability of conidia was determined on SDA-B. The bioassays were designed in two steps. First, the fungal isolates were tested for pathogenicity against mosquito adults. Bioassays were conducted in a plastic cylinder (height 18, diameter 9 cm) capped with netting material. To evaluate the pathogenicity of the fungus, 10 mosquito adults aged 1–2 days were placed in the cylinder, and 1 mL of a conidial suspension (1 × 10^8^ conidia/mL) was sprayed into each cylinder using an SD tower sprayer (Shin et al. 2011). Cotton wool freshly soaked with a 10% sucrose solution was placed on the netting. The control group was treated with the same method but without conidia. To determine whether the dead mosquitoes had been infected by the fungus, after 10 days of treatment, all the mosquito cadavers were removed from the cylinder and maintained at over 90% RH in an incubator at 25 ± 1 °C for 7 days. Cadavers with visible fungal growth on their body surface were considered to have died as a result of fungal infection. Additional bioassays were performed to determine the virulence of the fungal isolates. Twenty mosquitoes were used in the bioassay with the same methods described above. Mosquitoes were checked daily for mortality, and the bioassay was repeated three times.

### Conidia productivity

Conidia were produced in Petri dishes on Italian millet grains (*Setaria italica* Beauvois). Millet grains for the production of conidia were prepared according to a previous report (Kim et al. 2011). Briefly, millet grains were placed in a polyvinyl bag and soaked in a half volume of water containing 0.15% citric acid. The bag was held at 95 °C for 15 min and then autoclaved at 121 °C for 30 min. After cooling to ambient temperature, the millet grains (5 g) were divided into the Petri dishes and inoculated with a suspension of 1 × 10^7^ conidia (1 mL). The conidia on the millet grains were collected daily and counted using a haemocytometer.

### Statistical analysis

The mortality data were analysed by SPSS statistical software ver. 25.0 (SPSS, Inc., Chicago, IL, USA). The lethal time for the treatments was analysed with probit analysis. Data were expressed as the mean ± standard error (SE), and statistical significance was set at the conventional α < 0.05 level.

## Results

### Evaluation of thermotolerance and UV-B tolerance

Most of the 109 *B. bassiana* isolates used in this study showed low thermotolerance but high UV-B tolerance (Additional file 1: Table S1). The isolates with more than 50% thermotolerance accounted for 10.1% (11 isolates), and those with 30% or more accounted for only 43.1%. On the other hand, most of the fungal isolates showed more than 50% UV-B tolerance, and 67.9% of the isolates showed more than 70% tolerance. As most of the isolates had high UV-B tolerance, 30 isolates were selected based on thermotolerance and used for the evaluation of pathogenicity against the mosquito adults (Table 1).

**Table 1:** Thermotolerance and UV-B tolerance of *B. bassiana* isolates

*B. bassiana* isolates	Conidial germination (%)	
	Exposure to 45 °C for 2 h	Exposure to UV-B on 0.2 J
KG22R3W1	79.99	94.56
JB6G3W1	63.21	82.64
KW10S1W1	62.20	100.00
JN19M2W1	61.05	88.81
JN16R1W1	60.10	80.17
CN3R2W1	60.04	75.80
DK4T4W1	58.89	86.05
CB12M1W1	57.46	91.33
KN3S1W1	54.23	84.90
JN5R1W1	52.25	72.03
CB3S3W2	50.69	67.45
JB7R3W1	49.46	72.06
JB13S1W1	46.81	86.17
CN6T1W2	46.13	79.71
KB6S1W1	45.98	92.25
JB13G1W1	45.58	83.24
CN14S2W1	45.11	80.77
JN19M4W1	44.31	67.70
CB12G2W1	44.10	81.69
KN16G1W1	43.84	72.75
JN15T2W1	43.30	63.51
KN13S1W1	43.21	66.09
KG1G2W1	42.54	62.13
CN5R1W1	42.11	87.20
CN13R1W1	40.99	75.19
CN5G1W1	39.72	100.00
KG19S1W1	39.08	71.46
CN10G3W2	38.68	74.37
KG11G1W1	37.92	66.37
KW10G7W1	37.67	78.76

### Pathogenicity to mosquito adults

The pathogenicity of the fungi to *Ae. albopictus* and *Cx. pipiens* mosquito adults was examined using 30 isolates of *B. bassiana* that had a high thermotolerance and UV-B tolerance. The conidia of the selected isolates were applied to the two species of mosquitoes, and only the isolates that produced confirmed mycosis of the cadavers were considered pathogenic *B. bassiana* isolates. As a result, typical fungal symptoms were observed over time in dead mosquitoes after conidia treatment (Additional file 2: Figure S1). Twenty-three isolates were found to be pathogenic against *Ae. albopictus* and 12 isolates were pathogenic against *Cx. pipiens* adults (Table 2). Among the pathogenic isolates, 6 were determined to be simultaneously pathogenic to *Ae. albopictus* and *Cx. pipiens* adults.

**Table 2:** Pathogenicity of *B. bassiana* isolates against *Ae. albopictus* and *Cx. pipiens* mosquito adults

*B. bassiana* isolates	Pathogenicity	
	*Ae. albopictus*	*Cx. pipiens*
CB12G2W1	X	X
CB3S3W2	○	X
CB12M1W1	○	X
CN6T1W2	○	○
DK4T4W1	○	X
KW10G7W1	○	X
KW10S1W1	○	X
JB13G1W1	○	X
JB6G3W1	○	X
JB7R3W1	○	X
JB13S1W1	○	X
JN19M2W1	△	○
JN16R1W1	○	X
JN5R1W1	○	○
JN15T2W1	○	○
JN19M4W1	○	X
KG1G2W1	○	X
KG11G1W1	○	X
KN3S1W1	△	○
KN16G1W1	○	○
KN13S1W1	○	X
KG22R3W1	X	○
KG19S1W1	○	X
KB6S1W1	○	○
CN13R1W1	○	X
CN3R2W1	△	○
CN10G3W2	○	○
CN5R1W1	X	○
CN14S2W1	△	○
CN5G1W1	○	X

○: Showed death and mycosis; X: did not show death; △: showed death but did not mycosis

### Virulence in mosquito adults

The virulence of 6 isolates of *B. bassiana* that were simultaneously pathogenic to *Ae. albopictus* and *Cx. pipiens* adults was evaluated against each mosquito species. The pathogenicity of the 6 isolates was confirmed in both mosquito species; 4 isolates pathogenic to *Ae. albopictus* and 5 isolates pathogenic to *Cx. pipiens* showed 100% mortality at 10 days after treatment (Table 3). Moreover, 3 isolates of *B. bassiana* (CN6T1W2, JN5R1W1 and JN5T2W1) showed 100% mortality against both species of mosquito adults. Regarding lethality, all isolates were found to be slightly more pathogenic to *Ae. albopictus* than to *Cx. pipiens* adults. The LT_50_ values of *B. bassiana* CN6T1W2 and JN5R1W1 in *Cx. pipiens* were 4.3 and 4.4 days, respectively, which were lower than the values for the other 4 isolates, which were all more than 5.2 days. *B. bassiana* CN6T1W2 and JN5R1W1 also showed the lowest LT_50_ values for *Ae. albopictus* at 4.8 and 4.5 days, respectively. The LT_90_ values of *B. bassiana* CN6T1W2 and JN5R1W1 were also significantly lower than those of the other isolates. Thus, *B. bassiana* CN6T1W2 and JN5R1W1 were found to be the most virulent for both species of mosquitoes at the same time.

**Table 3:** Cumulative mortality ± standard error (SE) and lethal time (days), with respective confidence interval (CI) to kill 50 or 90% (LT_50_ and LT_90_) *Ae. albopictus* and *Cx. pipiens* adults treated with *B. bassiana* conidia (10^8^ conidia/mL)

Isolates	Mortality ± SE		Lethal time (CI)		Slope ± SE
	5 days	10 days	LT_50_	LT_90_	
*Ae. albopictus*					
CN6T1W2	58.3 ± 6.8 ^cd^	100^b^	4.8 (4.6–4.9)^a^	5.8 (5.5–6.2)^a^	14.76 ± 0.82
JN5R1W1	72.3 ± 10.1^d^	100^b^	4.5 (4.2–4.8)^a^	5.5 (5.2–6.2)^a^	14.41 ± 0.78
JN15T2W1	43.1 ± 7.7^bc^	100^b^	5.4 (5.1–5.8)^a^	7.5 (7–8.2)^ab^	9.19 ± 0.32
KN16G1W1	21.6 ± 2.8^ab^	100^b^	5.5 (3.6–7.7)^a^	8.5 (6.5–27.8)^ab^	6.91 ± 0.26
KB6S1W1	–	91.7 ± 8.3^b^	8.2 (7.7–8.8)^b^	11.3 (10.2–13.6)^b^	9.32 ± 0.43
CN10G3W2	13.3 ± 5.4^a^	68.3 ± 1.4^a^	9.2 (8.3–10.7)^b^	18.4 (14.7–27)^c^	4.29 ± 0.26
*F*_5,8_	15.87	6.28	–	–	–
*P*	0.001	0.012	–	–	–
*Cx. pipiens*					
CN6T1W2	76.5 ± 13.1^b^	100	4.3 (3.8–4.7)^a^	7.0 (6.3–8.2)^a^	6.03 ± 0.2
JN5R1W1	66.2 ± 4.5^b^	100	4.4 (3.7–5)^a^	6.2 (5.5–8.1)^a^	8.33 ± 0.28
JN15T2W1	34.9 ± 4.2^ab^	100	5.2 (4.8–5.5)^ab^	7.0 (6.5–7.7)^a^	9.92 ± 0.34
KN16G1W1	52.9 ± 12.5^ab^	90.5 ± 9.5	5.5 (4.8–6.1)^ab^	8.1 (7.2–9.9)^a^	7.54 ± 0.25
KB6S1W1	39.7 ± 15.3^ab^	100	5.2 (4.6–5.6)^ab^	7.9 (7–9.3)^a^	6.93 ± 0.23
CN10G3W2	13.6 ± 3.7^a^	100	6.3 (4.9–7.9)^b^	10.1 (8–20.4)^b^	6.15 ± 0.25
*F*_5,11_	3.95	0.91	–	–	–
*P*	0.027	0.511	–	–	–

Values, based on three repetitions, in the same column for each isolate followed by different letters were significantly different based on ANOVA and SNK test (cumulated mortalities; a–d) or their CI (LT; a–c). Cumulative control mortality was ≤ 5% at 10-days after treatment

### Conidia productivity on millet grain

**Fig. 1:**
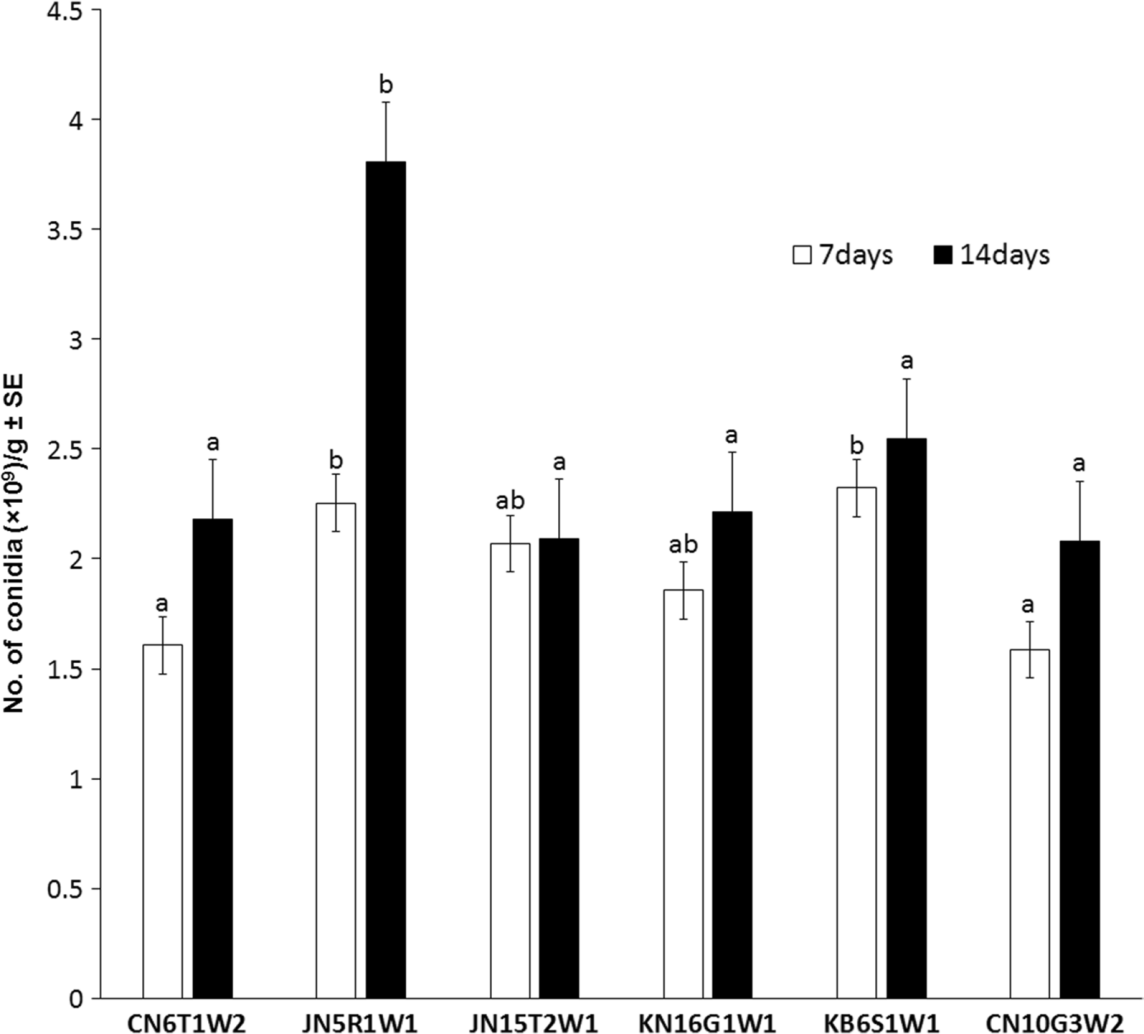
Productivity of *B. bassiana* conidia on millet grain medium. Conidia were sampled at 7 days and 14 days after inoculation of each *B. bassiana* isolate. Data are expressed as the mean ± standard error (SE). Values with different letters are significantly different (*p* < 0.05, SNK test in one-way ANOVA). All experiments were replicated three times

The conidia productivity of the 6 isolates of *B. bassiana* that were simultaneously pathogenic to the two species of mosquitoes was evaluated on millet grain medium. All the isolates showed conidia productivity of 1.6–2.3 × 10^9^ conidia/g at 7 days after inoculation; there was no significant difference in productivity (Fig. 1). At 14 days after inoculation, 5 isolates, excluding *B. bassiana* JN5R1W1, produced 2.1–2.5 × 10^9^ conidia/g, which was not significantly higher than that at 7 days. However, *B. bassiana* JN5R1W1 produced 3.8 × 10^9^ conidia/g at 14 days after inoculation, showing a very high increase in productivity compared with other isolates.

## Discussion

Finally, *B. bassiana* JN5R1W1 was selected as the most effective fungus for the simultaneous control of *Ae. albopictus* and *Cx. pipiens* adults and deposited in Korean Agricultural Culture Collection of National Institute of Agricultural Sciences with registration number of KACC83026BP. This fungal isolate showed high virulence against both species of mosquitoes and high conidia persistence and productivity. LT_50_ values by *B. bassiana* JN5R1W1 were 4.5 days (*Ae. albopictus*) and 4.4 days (*Cx. pipiens*). Although there are few report of entomopathogenic fungal virulence on *Ae. albopictus* and *Cx. pipiens* adults, similar report has shown that *M. anisopliae* showed 3.1–4.1 days against *Ae. aegypti* adult (Scholte et al. 2007). To the best of our knowledge, this is the first report about *B. bassiana* isolate that has dual virulence to *Ae. albopictus* and *Cx. pipiens* adults. The need for the simultaneous control of *Ae. albopictus* and *Cx. pipiens* is very high because they transfer different diseases, but their distribution areas overlap greatly (Khaiboullina et al. 2018; Kraemer et al. 2015; Lounibos 2002). Until now, the control methods for these mosquitoes have been largely dependent on chemical insecticides. However, the development of other control methods is urgent because of the various side effects of chemical insecticides (Ariee et al. 2001; Damalas and Eleftherohorinos 2011; Mužinić and Želježić 2018). Therefore, the research and development of various control methods with relatively low environmental impacts, such as entomopathogenic fungi, has attracted much attention. However, studies on fungi pathogenic to *Ae. albopictus* and *Cx. pipiens* are scarcer than studies on other mosquito species. Much of the fungi research has been conducted for the control of *Ae. aegypti* or *Anopheles* spp., and there have been many reports on various fungi, including *B. bassiana* and *M. anisopliae,* for these mosquito species (Huang et al. 2017; Scholte et al. 2004). There have been some reports of *M. anisopliae* for the larval and adult control of *Ae. albopictus* and *B. bassiana* for adult control using a contamination device, with remarkable results (Buckner et al. 2017; Snetselaar et al. 2014). In the case of *Cx. pipiens*, the research is relatively limited and only fungal metabolites were considered for larval control (Weiser et al. 1992). Eco-friendly insecticides are mainly used for the control of mosquito larvae, and effective products such as Bti and *L. sphaericus* have been developed and widely used. However, since the recently developed contamination device has been successful in the control of adult mosquitoes (Buckner et al. 2017; Snetselaar et al. 2014), the selection of a fungus to control mosquito adults is considered to be important. Although mosquito control using fungi has been considered, the fact that only one contamination device has been produced may be due to the ineffective methods of spraying fungal conidia for mosquito adults and treating water containing mosquito larvae. Therefore, it is considered that a treatment method similar to that of the contamination device is the most efficient at present; thus, the proper selection of a fungus to be utilized with this treatment method is becoming important. The fungal isolate selected in this study is expected to be effective for the simultaneous control of *Ae. albopictus* and *Cx. pipiens* adults.

In addition, the results of our study showed that 12 isolates and 23 isolates of *B. bassiana* were pathogenic to *Cx. pipiens* and *Ae. albopictus*, respectively, and *Ae. albopictus* was more susceptible to *B. bassiana*. However, until now, the pathogenicity of some *M. anisopliae* (Scholte et al. 2007) and *B. bassiana* strains (Buckner et al. 2017) has been tested on *Ae. albopictus* adults, but the pathogenicity of *M. anisopliae* and *B. bassiana* has not been reported for *Cx. pipiens* adults. Therefore, further research on the susceptibility of these mosquito species to *B. bassiana* using more isolates is needed. In particular, only 6 isolates of *B. bassiana* were found to be simultaneously pathogenic to these two species of mosquitoes; reports of a fungus that is simultaneously pathogenic to two or more species of mosquitoes are rare. Similar studies have reported simultaneous pathogenicity in *Ae. aegypti* and *Ae. Albopictus*, which are of the same genus (Buckner et al. 2017; Scholte et al. 2007). However, there have been no reports on the selection of fungi that are simultaneously pathogenic to two species of mosquitoes from different genera.

In our study, we attempted to increase the industrial usefulness of selected fungi by using only fungal isolates that are expected to be highly persistent in the natural environment. Traditionally, the selection of entomopathogenic fungi for the development of insecticides has focused mainly on their virulence to target insects. However, in terms of industrial usefulness, the persistence of fungicidal agents when applied in outdoor environments or during the distribution process is just as important as virulence (Fernandes et al. 2015; Lee et al. 2015). Therefore, in recent years, there have been an increasing number of attempts to evaluate various factors simultaneously and to select suitable fungi for control. Many environmental factors affect the persistence of fungi, but the most significant effects are thought to be thermotolerance and UV-B tolerance (Fernandes et al. 2008, 2015; Lee et al. 2015).

Thermotolerance is closely related to the production of reactive oxygen species (ROS) (Zhang et al. 2017) and heat shock proteins (HSPs) (Xie et al. 2010). Thermotolerance could be enhanced by the overexpression of HSP25 and pyruvate kinase or superoxide dismutase (Wu et al. 2019; Xie et al. 2010). In addition, it is possible to increase the thermotolerance of fungus by controlling the nutrients or the osmotic pressure of the medium (Rangel et al. 2015). Such medium conditioning could also enhance UV tolerance. UV tolerance is related to the conidial colour of fungus; darkly pigmented conidia are more tolerant to UV radiation than less-pigmented conidia (Rangel et al. 2006). However, it was also reported that conidial pigmentation may be important to protect from UV radiation, but it is not crucial (Fernandes et al. 2015). In our results, although *B. bassiana* with white conidia was used, most of the isolates showed high UV-B tolerance with more than 50%. UV-tolerance can be assessed with various UV doses, but we set the standard of 0.2 J/cm^2^ for various tolerances of fungi according to the previous report (Fernandes et al. 2015). These various manipulations and controls may enhance the tolerance of fungus, but they may have limitations if the selected fungus does not have high thermotolerance. Therefore, it is important to select and use a fungus with high thermotolerance. In the present study, only *B. bassiana* isolates with high thermotolerance and UV-B tolerance were used, so the practical use of the finally selected isolate is be expected to be high.

To develop appropriate fungal insecticides, virulence to the target insect, conidia persistence and conidia productivity are important factors (Lopez-Perez et al. 2015; Muñiz-Paredes et al. 2017). Recently, several studies have been conducted on various media that can increase the conidial productivity of fungi (Lopez-Perez et al. 2015; Kim et al. 2014; Safavi et al. 2007; Shah et al. 2005). However, when the basic conidia productivity of the fungus is low, the replacement of the medium is a limitation. Therefore, a comparative evaluation of the conidia productivity of fungi is also important for proper fungus selection. The productivity of conidia as an evaluation factor for the selection of the final fungal isolates was added as a selection index in our study. Conidia productivity was evaluated on millet grain medium, which was reported to be effective for the production and tolerance of conidia (Kim et al. 2011). The conidia productivity of *B. bassiana* in various solid media was reported with approximately 1.0–2.9 × 10^10^ conidia/g on dry substrate, but it was approximately 1.8–2.2 × 10^9^ conidia/g on wet substrate as our same condition (Lopez-Perez et al. 2015). Additionally, the conidia productivity in the Italian millet medium used in our study was reported to be approximately 1.5–2.5 × 10^9^ conidia/g (Song et al. 2019). This indicated that our finally selected *B. bassiana* JN5R1W1 has the high conidia productivity with 3.8 × 10^9^ conidia/g. The difference in conidia productivity between the fungal isolates in our study indicated that this comparison was necessary to select the optimal fungal isolate. Although our study used only millet grain and evaluated only conidia productivity, it may be beneficial to perform further studies on the virulence and tolerance of fungal conidia produced on various media.

In conclusion, *B. bassiana* JN5R1W1 was finally selected for the simultaneous control *Ae. albopictus* and *Cx. pipiens* adults through evaluations of virulence, persistence and productivity of the fungal conidia. The development of successful fungal insecticides containing this fungal isolate for the simultaneous control of two species of mosquitoes is expected in further studies on optimal formulations and field tests.

## Supplementary information

**Additional file 1: Table S1.** Thermotolerance and UV-B tolerance of *Beauveria bassiana* isolates from Korea.

**Additional file 2: Figure S1.** Cadavers of *Ae. albopictus* (A) and *Cx. pipiens* (B) adults showing sporulation of *B. bassiana*.

## Data Availability

All data generated during this study are included in this article and its additional files.

## Abbreviations

UV: ultraviolet
RH: relative humidity
PDA: potato dextrose agar
SDA: Sabouraud dextrose agar
SDA-B: Sabouraud dextrose agar with benomyl
LT_50_: the median lethal time
LT_90_: the 90% lethal time
