# Supplementary material for: Beauveria bassiana for the simultaneous control of Aedes albopictus and Culex pipiens mosquito adults shows high conidia persistence and productivity
Source: AMB Express. 2019 Dec 21;9:206. doi: 10.1186/s13568-019-0933-z (PMC6925604; doi:10.1186/s13568-019-0933-z)
Supplement: Supplementary file 1 — Additional file 1: Table S1. Thermotolerance and UV-B tolerance of Beauveria bassiana isolates from Korea. [file 13568_2019_933_MOESM1_ESM.docx]

**Table S1 Thermotolerance and UV-B tolerance of *B. bassiana* isolates from Korea**

| ***B. bassiana* isolates** | **Conidial germination rate (%)** | |
| --- | --- | --- |
|  | **Exposure to 45 ℃ for 2 h** | **Exposure to UV-B on 0.2 J** |
| KG22R3W1 | 79.99 | 94.56 |
| JB6G3W1 | 63.21 | 82.64 |
| KW10S1W1 | 62.20 | 100.00 |
| JN19M2W1 | 61.05 | 88.81 |
| JN16R1W1 | 60.10 | 80.17 |
| CN3R2W1 | 60.04 | 75.80 |
| DK4T4W1 | 58.89 | 86.05 |
| CB12M1W1 | 57.46 | 91.33 |
| KN3S1W1 | 54.23 | 84.90 |
| JN5R1W1 | 52.25 | 72.03 |
| CB3S3W2 | 50.69 | 67.45 |
| JB7R3W1 | 49.46 | 72.06 |
| JB13S1W1 | 46.81 | 86.17 |
| CN6T1W2 | 46.13 | 79.71 |
| KB6S1W1 | 45.98 | 92.25 |
| JB13G1W1 | 45.58 | 83.24 |
| CN14S2W1 | 45.11 | 80.77 |
| JN19M4W1 | 44.31 | 67.70 |
| CB12G2W1 | 44.10 | 81.69 |
| KN16G1W1 | 43.84 | 72.75 |
| JN15T2W1 | 43.30 | 63.51 |
| KN13S1W1 | 43.21 | 66.09 |
| KG1G2W1 | 42.54 | 62.13 |
| CN5R1W1 | 42.11 | 87.20 |
| CN13R1W1 | 40.99 | 75.19 |
| CN5G1W1 | 39.72 | 100.00 |
| KG19S1W1 | 39.08 | 71.46 |
| CN10G3W2 | 38.68 | 74.37 |
| KG11G1W1 | 37.92 | 66.37 |
| KW10G7W1 | 37.67 | 78.76 |
| CB2T1W1 | 37.35 | 82.08 |
| CB12S2W1 | 36.93 | 74.21 |
| CN9G1W1 | 36.37 | 66.96 |
| KW3G10W1 | 36.34 | 50.95 |
| KN18R1W1 | 35.85 | 69.20 |
| JN10S2W1 | 35.58 | 62.37 |
| KB20T1W1 | 34.89 | 75.58 |
| JN12S3W1 | 34.21 | 79.38 |
| JB12G1W1 | 33.20 | 78.90 |
| CB6S1W1 | 33.17 | 93.70 |
| KG22F2W1 | 32.70 | 87.34 |
| JN13S3W1 | 32.20 | 72.69 |
| JN13M1W1 | 32.05 | 59.19 |
| KW2S2W2 | 31.31 | 69.19 |
| CN10G2W1 | 31.30 | 85.57 |
| CN4T2W1 | 30.47 | 68.84 |
| KB9R2W2 | 30.27 | 52.34 |
| CN13G1W1 | 29.68 | 74.79 |
| CN9G1W2 | 29.36 | 69.53 |
| CN6T1W1 | 28.46 | 91.92 |
| JN15R2W1 | 28.32 | 68.14 |
| JN4G1W1 | 26.61 | 77.29 |
| KN18G1W1 | 25.95 | 92.18 |
| JN13S5W1 | 24.87 | 77.83 |
| KB22G6W1 | 23.81 | 90.92 |
| JB5F1W1 | 22.41 | 61.61 |
| KW9G3W1 | 22.40 | 63.78 |
| KB21T3W1 | 21.88 | 68.17 |
| CB11S1W1 | 21.20 | 94.25 |
| KB11G2W2 | 20.90 | 59.01 |
| KG22F1W1 | 20.85 | 92.95 |
| KW7M2W1 | 18.99 | 56.32 |
| KB6S1W2 | 17.36 | 46.44 |
| KW7S5W1 | 17.04 | 58.49 |
| KW4T15W1 | 16.95 | 68.24 |
| KW12G1W1 | 16.42 | 49.94 |
| DK3T3W1 | 16.15 | 86.68 |
| KB5G2G1 | 15.59 | 87.74 |
| KW2T1W1 | 15.15 | 48.98 |
| KW18S1W1 | 14.42 | 58.42 |
| KN3F1W1 | 13.36 | 72.80 |
| KW7S3W2 | 11.69 | 80.94 |
| JN5T7W1 | 11.01 | 82.78 |
| KB12G1W1 | 10.98 | 67.09 |
| CB12M7W1 | 10.70 | 89.71 |
| KW8S3W1 | 10.15 | 58.90 |
| KW4G2W1 | 9.90 | 77.36 |
| CB7S2W2 | 9.84 | 99.88 |
| JN19M6W1 | 6.85 | 86.66 |
| KW17T3W1 | 6.07 | 91.36 |
| CN16S3W1 | 5.26 | 59.61 |
| KB21G4W2 | 5.01 | 63.16 |
| JN1T1W1 | 4.98 | 87.03 |
| KB14G2W1 | 4.58 | 50.09 |
| JN13S1W1 | 4.13 | 66.61 |
| KB21G2W1 | 3.97 | 59.87 |
| KB13M1W1 | 3.87 | 76.94 |
| KB22M2W2 | 3.29 | 93.28 |
| KW9F2W1 | 3.26 | 76.36 |
| CN3T1W1 | 3.10 | 84.76 |
| CB12M2W1 | 3.09 | 61.10 |
| KB12S2W1 | 3.08 | 82.12 |
| KB13M2W1 | 2.74 | 55.72 |
| KB9R2W1 | 2.70 | 68.02 |
| CB8M8W1 | 2.49 | 76.38 |
| CN10G3W1 | 1.83 | 77.27 |
| CB12M5W1 | 1.10 | 74.47 |
| KW9R1W1 | 1.08 | 76.66 |
| KW7S1W1 | 1.06 | 88.10 |
| JB3S1W1 | 0.97 | 75.06 |
| KG19T2W1 | 0.84 | 73.50 |
| KB17S1W2 | 0.65 | 67.06 |
| DK1R1W1 | 0.58 | 34.90 |
| CN5R2W1 | 0.55 | 72.42 |
| KB22M2W1 | 0.35 | 74.29 |
| KW5S1W1 | 0.23 | 64.84 |
| CB7S2W1 | 0.12 | 66.59 |
| KW9G4W2 | 0.00 | 71.99 |
| KB20S1W1 | 0.00 | 67.15 |
| KB17S1W1 | 0.00 | 64.24 |
| KW4G1W1 | 0.00 | 62.00 |
| KB4G2W1 | 0.00 | 69.41 |
| CB7S1W1 | 0.00 | 51.12 |
| KB11S2W1 | 0.00 | 42.84 |
| KB21T1W2 | 0.00 | 81.28 |
| KB21T1W1 | 0.00 | 64.88 |
| KW16T2W1 | 0.00 | 86.63 |
| KB22M1W1 | 0.00 | 69.69 |
| KN19M1W1 | 0.00 | 72.13 |
| KW12T1W1 | 0.00 | 67.36 |
| KB15R1W1 | 0.00 | 87.27 |
| KW5G1W1 | 0.00 | 60.60 |
| KW7S3W1 | 0.00 | 68.57 |
| KB14M1W1 | 0.00 | 78.10 |
| JB9S1W1 | 0.00 | 58.90 |
| KG7R2W1 | 0.00 | 78.28 |
| JB9M1W1 | 0.00 | 59.73 |
| JB21G1W1 | 0.00 | 96.74 |
