# Supplementary figures and images for: Beauveria bassiana for the simultaneous control of Aedes albopictus and Culex pipiens mosquito adults shows high conidia persistence and productivity
Source: AMB Express. 2019 Dec 21;9:206. doi: 10.1186/s13568-019-0933-z (PMC6925604; doi:10.1186/s13568-019-0933-z)

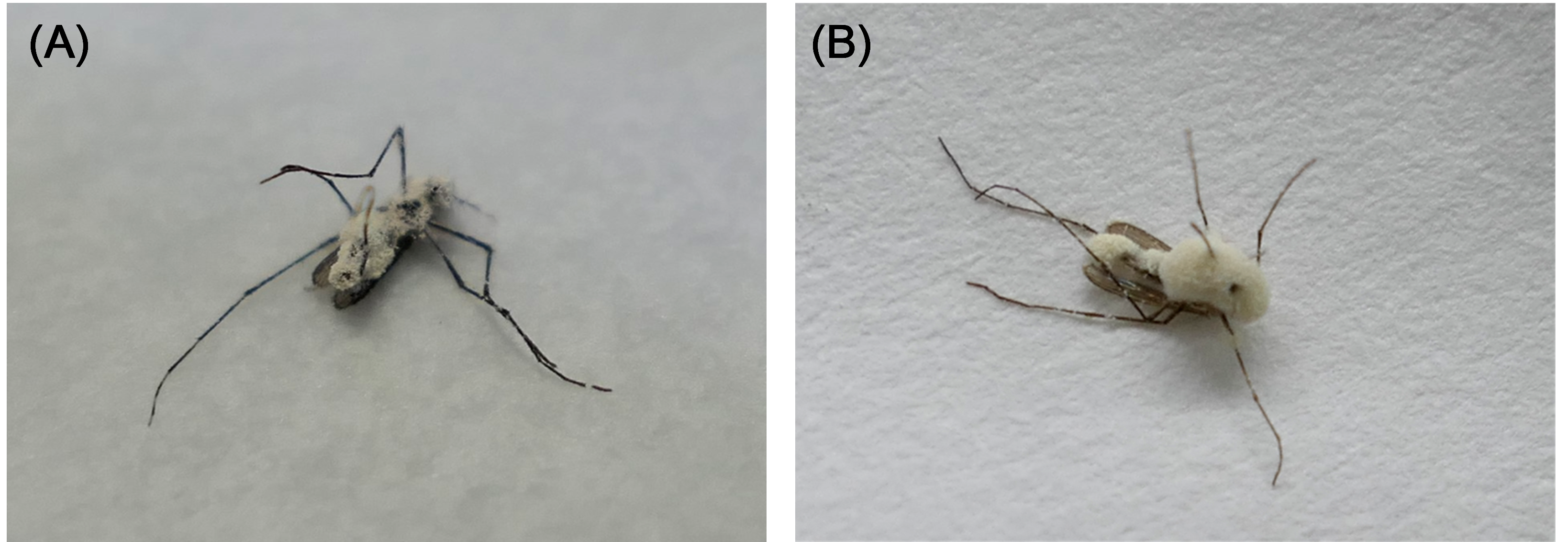

Supplement: Supplementary file 2 — Additional file 2: Figure S1. Cadavers of Ae. albopictus (A) and Cx. pipiens (B) adults showing sporulation of B. bassiana. [file 13568_2019_933_MOESM2_ESM.tif]
